# Perception and practices of depth of anesthesia monitoring and intraoperative awareness event rate among Jordanian anesthesiologists: a cross-sectional study

**DOI:** 10.1186/s12871-022-01941-w

**Published:** 2022-12-27

**Authors:** Sager Nawafleh, Ahmad Alrawashdeh, Omar Ababneh, Morad Bani-Hani, Zaid Al Modanat, Diab Bani Hani, Adel Bataineh, Faisal Al-Salameh, Sajeda Abuzaid, Omer Yasser, Khaled Khairallah

**Affiliations:** 1 Department of General Surgery, Urology and Anesthesia, Faculty of Medicine, The Hashemite University, Zarqa, 13115 Jordan; 2 Department of Allied Medical Science, Faculty of Applied Medical Science, Jordan University of Science and Technology, Irbid, 22110 Jordan; 3 Department of Anesthesia and Intensive Care, School of Medicine, The University of Jordan, Amman, 11942 Jordan; 4 Faculty of Medicine, Yarmouk University, Irbid, 21110 Jordan; 5 Department of Anesthesia and Recovery, Faculty of Medicine, Jordan University of Science and Technology, Irbid, 21110 Jordan; 6 Anesthesia Department, Istiklal Hospital, Amman, Jordan; 7 Department of Research and Data Analytics, Kernel Center, Irbid, 21110 Jordan; 8 Department of Public Health and Community Medicine, Faculty of Medicine, Jordan University of Science and Technology, Irbid, 22110 Jordan

**Keywords:** Depth of anesthesia monitors, Intraoperative awareness, Perception, Practice, Jordan

## Abstract

**Background:**

Intraoperative awareness is the second most common complication of surgeries, and it negatively affects patients and healthcare professionals. Based on the limited previous studies, there is a wide variation in the incidence of intraoperative awareness and in the practices and attitudes toward depth of anesthesia (DoA) monitoring among healthcare systems and anesthesiologists. This study aimed to evaluate the Jordanian anesthesiologists’ practice and attitudes toward DoA monitoring and estimate the event rate of intraoperative awareness among the participating anesthesiologists.

**Methods:**

A descriptive cross-sectional survey of Jordanian anesthesiologists working in public, private, and university hospitals was utilized using a questionnaire developed based on previous studies. Practice and attitude in using DoA monitors were evaluated. Anesthesiologists were asked to best estimate the number of anesthesia procedures and frequency of intraoperative awareness events in the year before. Percentages and 95% Confidence Intervals (95%CI) were reported and compared between groups using chi-square tests.

**Results:**

A total of 107 anesthesiologists responded and completed the survey. About one-third of the respondents (34.6%; 95% CI 26.1–44.2) had never used a DoA monitor and only 6.5% (95% CI 3.1–13.2) reported using it as a “daily practice”. The use of a DoA monitor was associated with experience and type of health sector. However, 81.3% (95% CI 66.5–83.5) believed that currently available DoA monitors are effective for DoA monitoring and only 4.7% (95%CI 1.9–10.8) reported it as being “invalid”. Most respondents reported that the main purpose of using a DoA monitor was to prevent awareness (86.0%; 95%CI 77.9–91.4), guide the delivery of anesthetics (63.6%; 95%CI 53.9–72.2), and reduce recovery time (57%; 95%CI 47.4–66.1). The event rate of intraoperative awareness was estimated at 0.4% among participating anesthesiologists. Most Jordanian hospitals lacked policy intending to prevent intraoperative awareness.

**Conclusions:**

Most anesthesiologists believed in the role of DoA monitors in preventing intraoperative awareness, however, their attitudes and knowledge are inadequate, and few use DoA monitors in routine practices. In Jordan, large efforts are needed to regulate the use of DoA monitoring and reduce the incidence of intraoperative awareness.

## Background

Intraoperative awareness is a terrifying sequela of anesthetic procedures and is characterized as an unexpected recall of patient consciousness during surgical procedures occurring when general anesthesia is not maintained or achieved [1]. The incidence rate of intraoperative awareness was estimated between 1–2 for ervery1000 cases in general patients and 1/100 in high-risk patients [2–4]. This is considered clinically significant given millions of general anesthesia performed annually around the world and its negative effects on patients, healthcare providers, and hospitals. For example, more than 70% of patients who experienced intraoperative awareness reported suffering from post-traumatic stress disorder (PTSD) [5]. Furthermore, intraoperative awareness is within the top three causes of legal actions taken against hospitals [1]. To improve healthcare quality and maintain patient safety, it is crucial to track intraoperative awareness and evaluate anesthesiologists’ practices and attitudes toward depth of anesthesia (DoA) monitoring.

Factors contributing to the occurrence of awareness incidents can be classified into patient conditions (i.e. history of cardiopulmonary diseases, alcohol use or smoking), [6] surgery characteristics (i.e. type and duration of surgery), and anesthesiologists’ practices on monitoring DoA [7–11]. Currently, two techniques are used to monitor the DoA, including observing clinical features such as heart rate, blood pressure, movement, sweating, and using an electroencephalogram (EEG) [1]. Monitoring clinical signs was seen to be insufficient in preventing intraoperative awareness [12]. Using new indexes of the EEG signal, such as bispectral index (BIS), were suggested to reduce the risk of intraoperative awareness [12–15]. However, other studies have failed to demonstrate the superiority of these monitors in preventing intraoperative awareness compared to clinical surveillance or end-tidal anesthesia concentration (ETAC) [16–18]. These findings may explain the variation in the practice and attitudes of anesthesiologists toward using DoA monitoring.

Few research studies assessed anesthesiologists’ practices and attitudes toward awareness and depth of anesthesia monitoring. In Australia, only 29% of anesthesiologists used DoA monitoring in more than one-third of cases, and 30% of anesthesiologists believed in the effectiveness of using a DoA monitor in preventing intraoperative awareness [19]. In China, only 9.1% of anesthesiologists routinely used DoA monitors, and 65% of respondents believed that DoA monitors should combine EEG and vital signs monitoring [20]. To the best of our knowledge, there is a lack of studies evaluating the practice and perception of anesthesiologists in developing countries, including the Middle East region. The objective of this study was to investigate the perception, attitudes, and current practices of Jordanian anesthesiologists on DoA monitoring and estimate the event rate of intraoperative awareness among them.

## Methods

### Study design and setting

This study was a descriptive cross-sectional survey of Jordanian anesthesiologists working in public, private, and university hospitals in Jordan, between May and August 2022, using an electronic form created on Kobo toolbox website. Institutional Review Board (IRB) approvals were obtained from Hashemite University (IRB No:23/6/2021/2022). This study has been performed in accordance with the Declaration of Helsinki.

### Study participants

We included a convenient sample of anesthesiologists regardless of their degree of education, specialty (residents, specialists, and consultants), and workload. We used the formula of Kizito, 2016 to estimate a representative sample size (*n* = N/[1 + N*(e)^2], where N refers to the total population and e refers to the margin of error) [21]. While the total population of anesthesiologists was estimated at around 1,000 and by using the margin of error of 5%, the required sample size estimation was 285 anesthesiologists. The survey link was sent to a total of 300 anesthesiologists through emails and private mobiles obtained from the major public and private hospitals and universities in Jordan. Snowballing technique was also used by asking the anesthesiologists to forward our survey to other anesthesiologists. Participation in this study was voluntary and no re-identified data were collected for confidentiality.

### Study survey and variables

The study survey was developed based on previous surveys conducted in the United Kingdom and China [1, 20]. The survey consisted of three main sections. Section one included 7 items (Table 1) for the demographic characteristics, covering age, gender, education, experience, and health sector. Section two included 24 items (Tables 2 and 3) concerning anesthesiologists’ practice and perception toward DoA monitoring and the use of a DoA monitor [20]. In section three, the rate and types of events of intraoperative awareness and their characteristics (Table 4) occurred in all anesthesia procedures during the year before (2021). We also asked whether their hospitals had policies for the prevention or management of intraoperative awareness. The types of the included questions and scales varied in this survey. The validity of the used survey was assessed previously using face validity, content validity, and construct validity. However, as a few changes had been made to the original surveys to comply with the local setting and healthcare system of Jordan, the face validity of our final survey was verified by five experienced anesthesiologists and researchers.

**Table 1:** Demographic characteristics of the respondents

	**Total**	**DoA monitor usage**		
	***n* (%)**	**Used *n* (%)**	**Never used *n* (%)**	***p* value**
**Total**	107 (100)	70 (65.4)	37(34.6)	
**Gender**				0.76
Male	91 (85.1)	59 (64.8)	32 (35.2)	
Female	16 (15.0)	11 (68.8)	5 (31.3)	
**Age groups, in years**				0.096
24–30	33 (30.8)	18 (54.6)	15 (45.5)	
30–39	55 (51.4)	36 (65.5)	19 (34.6)	
40–65	19 (17.8)	16 (84.2)	3 (15.8)	
**Education level**				0.003
Bachelor	62 (57.9)	33 (53.2)	29 (46.8)	
Master	34 (31.8)	26 (76.5)	8 (23.5)	
PhD	11 (10.3)	11 (100)	0 (0)	
**Specialty category**				0.002
Anesthesia consultant	16 (15.0)	14 (87.5)	2 (12.5)	
Anesthesia specialist	27 (25.2)	23 (85.2)	4 (14.8)	
Senior resident	43 (40.2)	24 (55.8)	19 (44.2)	
Junior resident	21 (19.6)	9 (42.9)	12 (57.1)	
**Health sector**				< 0.001
Ministry of Health	39 (36.5)	17 (43.6)	22 (56.4)	
Private hospital	32 (29.9)	28 (87.5)	4 (12.5)	
University hospital	36 (33.6)	25 (69.4)	11 (30.6)	
**Experience in years**				0.004
< 4	64 (59.8)	35 (54.7)	29 (45.3)	
≥ 5	43 (40.2)	35 (81.4)	8 (18.6)	
**Clinical work hours per day**				0.5
≤ 8 h/day	82 (76.6)	55 (67.1)	27 (32.9)	
> 8 h/day	25 (23.4)	15 (60)	10 (40)

**Table 2:** Perception of anesthesiologist toward the DoA monitor

	**Total respondents**		**Familiarity with DoA monitors**		***p* value**
	***n***	**% (95% CI)**	**Familiar 75 (70.1%)**	**Unfamiliar 32 (29.9%)**	
**Effectiveness of DoA monitors**					< 0.001
Very effective	55	51.4 (41.9–60.8)	40 (53.3)	15 (46.9)	
Little effective	32	29.9 (21.9–39.3)	30 (40.0)	2 (6.3)	
Invalid	5	4.7 (1.9–10.8)	2 (2.7)	3 (9.4)	
Unknown	15	14 (8.6–22.1)	3 (4.0)	12 (37.5)	
**Value of DoA monitor**					0.26
Accuracy	34	31.8 (23.6–41.3)	20 (26.7)	14 (43.8)	
Stability	33	30.8 (22.7–40.3)	23 (30.7)	10 (31.3)	
Cost-effectiveness	23	21.5 (14.7–30.4)	18 (24.0)	5 (15.6)	
Applicability	17	15.9 (10.1–24.2)	14 (18.7)	3 (9.4)	
**Purposes of using a DoA monitor in clinical practice**					
Preventing awareness	92	86 (77.9–91.4)	66 (88.0)	26 (81.3)	0.357
Guiding the delivery of anesthetics	68	63.6 (53.9–72.2)	50 (66.7)	18 (56.3)	0.305
Reducing recovery time	61	57 (47.4–66.1)	45 (60.0)	16 (50.0)	0.34
Avoiding deep anesthesia	53	49.5 (40.1–59)	43 (57.3)	10 (31.3)	0.013
Preventing side effects of anesthetics	39	36.5 (27.8–46.1)	27 (36)	12 (37.5)	0.9
Determining the cause of changes in hemodynamics	30	28 (20.3–37.4)	20 (26.7)	10 (31.3)	0.63
**Effectiveness of DoA monitors**					
General anesthesia with tracheal intubation	76	71 (61.6–78.9)	54 (72.0)	22 (68.8)	0.73
All general anesthesia	68	63.6 (53.9–72.2)	45 (60.0)	23 (71.9)	0.24
General anesthesia with spontaneous breathing	37	34.6 (26.1–44.2)	30 (40.0)	7 (21.9)	0.071
IV anesthesia for painless diagnosis and treatment	23	21.5 (14.7–30.4)	19 (25.3)	4 (12.5)	0.14
Local anesthesia with sedation	19	17.8 (11.6–26.3)	14 (18.7)	5 (15.6)	0.71
**Age-groups suitable for DoA**					
Elderly	70	65.4 (55.8–73.9)	44 (58.7)	26 (81.3)	0.025
Adults	70	65.4 (55.8–73.9)	51 (68.0)	19 (59.4)	0.39
Youth	60	56.1 (46.5–65.3)	44 (58.7)	16 (50.0)	0.4
Young children and infants	33	30.8 (22.7–40.3)	23 (30.7)	10 (31.3)	0.9

**Table 3:** Characteristics and differences in DoA monitoring practice between those who had a case of intraoperative awareness and those who did not

	**Total sample**		**Had a case of intraoperative awareness**		
	***n***	**% (95% CI)**	**Yes *n* (%)**	**No/unknown *n* (%)**	***p* value**
**DoA monitor Used**					
BIS	60	56.1 (46.5–65.3)	32 (56.1)	28 (56.0)	0.99
AEP	14	13.1 (7.9–21.0)	11 (19.3)	3 (6.0)	0.042
PSI	9	8.4 (4.4–15.5)	3 (5.3)	6 (12.0)	0.21
CSI	4	3.7 (1.4–9.6)	2 (3.5)	2 (4.0)	0.89
Narcotrend	3	2.8 (0.9–8.4)	0 (0)	3 (6.0)	0.061
Entropy	2	1.9 (0.5–7.3)	1 (1.8)	1 (2.0)	0.93
Never used	37	34.6 (26.1–44.2)	19 (33.3)	18 (36.0)	0.77
**Most accurate DoA monitor**					0.09
BIS	71	66.4 (56.8–74.7)	38 (66.7)	33 (66.0)	
AEP	14	13.1 (7.9–21.0)	8 (14.0)	6 (12.0)	
Other types	8	7.5 (3.8–14.3)	6 (10.5)	2 (4.0)	
CSI	6	5.6 (2.5–12.0)	2 (3.5)	4 (8.0)	
PSI	3	2.8 (0.9–8.4)	3 (5.3)	0 (0)	
Narcotrend	3	2.8 (0.9–8.4)	0 (0)	3 (6.0)	
Entropy	2	1.9 (0.5–7.3)	0 (0)	2 (4.0)	
**Most valuable indicator**					0.99
The number	21	19.6 (13.1–28.3)	11 (19.3)	10 (20.0)	
EEG trace	18	16.8 (10.8–25.2)	10 (17.5)	8 (16.0)	
Burst suppression ratio	12	11.2 (6.4–18.8)	6 (10.5)	6 (12.0)	
The three indicators are equally important	56	52.3 (42.8–61.7)	30 (52.6)	26 (52.0)	
**Proportion using a DoA monitor**					0.32
Always	7	6.5 (3.1–13.2)	4 (7.0)	3 (6.0)	
1/3 ~ 2/3	13	12.2 (7.1–19.9)	9 (15.8)	4 (8.0)	
< 1/3	72	67.3 (57.8–75.6)	34 (59.7)	38 (76.0)	
Never	15	14.0 (8.6–22.1)	10 (17.5)	5 (10.0)	

*DoA* Depth of anesthesia, *BIS* Bispectral index, *AEP* Auditory evoked potential, *PSI* Patient state index, *CSI* Cerebral state index, *EEG* electroencephalogram

**Table 4:** Characteristics of intraoperative awareness events

	**Frequency**	**Percentage (95% CI)**
Reports Volunteered by patient	57	25.2 (19.7–31.4)
Ascertained only on questioning	85	37.6 (31.3–44.3)
Awareness before surgery	75	33.2 (27.1–39.7)
Awareness during surgery	130	57.5 (50.8–64.1)
Awareness after surgery and before emergence	65	28.8 (23–35.1)
Physical or psychological hurt	40	17.7 (13–23.3)
Formal complaint to hospital	11	4.9 (2.5–8.5)

### Statistical analysis

Data were analyzed using STATA statistical package (STATA 16). A *p*-value of < 0.05 was considered statistically significant. For descriptive statistics, continuous variables were presented as means and standard deviations (SDs) and were compared between study groups using a student t-test or one-way analysis of variance (ANOVA), as appropriate. Whereas categorical variables were presented as frequencies and percentages and were compared between study groups using a chi-square test. We estimated the logit-transferred 95% confidence intervals for proportions. The rate of the intraoperative awareness events was estimated by dividing the total number of reported intraoperative awareness events for all participated anesthesiologists in 2021 by the total number of anesthesia procedures they performed during the same year.

## Results

A total of 107 physicians participated and completed the survey with a response rate of 35.7%. The mean age was 34.1 (SD ± 8.1) and 85.1% were male. Most of the respondents had a bachelor’s degree (57.9%) and were residents (59.8%). Among the respondents, about one-third of the participant (34.6%; 95%CI 26.1–44.2) had never used a DoA monitor. Similarly, a total of 29.9% (95%CI 21.9–39.3) of our sample were unfamiliar with DoA monitors. Of all respondents, a percentage of 53.3% (95% CI 43.7–62.6) had reported at least one event of intraoperative awareness during 2021, while 36.5% (95% CI 27.8–46.1) had not reported any event of intraoperative awareness, and 10.3 (95% CI 5.7–17.7) did not know if they had any event of intraoperative awareness.

Table 1 shows the difference in the demographic characteristics between those who had used and had never used a DoA monitor. The frequency of using a DoA monitor significantly differed by education level, specialty category, health sector, and experience in years.

Table 2 presents the perception of the respondents toward DoA monitors on their familiarity with the DoA monitors. Most of the respondents (81.3%; 95%CI.66.5–83.5) believed that currently available DoA monitors are effective for DoA monitoring, and only 4.7% (95%CI 1.9–10.8) of the respondents thought that they were invalid (Table 2). Respondents who were familiar with DoA monitors were more likely to believe that currently, available DoA monitors are effective, compared to those who were unfamiliar with DoA monitors (93.3% vs 53.2%; *p* < 0.001, respectively). Accuracy (31.8%; 95%CI 23.6–41.3) and stability (30.8%; 95%CI 22.7–40.3) were the most reported values of using a DoA monitor. Most respondents believed that the main purposes of using a DoA monitor were to prevent awareness (86.0%; 95%CI 77.9–91.4), guide the delivery of anesthetics (63.6%; 95%CI 53.9–72.2), and reduce recovery time (57%; 95%CI 47.4–66.1). More than half of the respondents believed that the anesthesia methods that are suitable for DoA monitoring are general anesthesia with endotracheal intubation (71%; 95%CI 61.6–78.9), followed by all general anesthesia (63.6%; 95%CI 53.9–72.2). Additionally, regarding the population for which DoA monitoring is applicable, approximately 65.7% (95%CI 55.8–73.9) of the respondents reported that old and adult patients are most in need of DoA monitoring. Surprisingly, compared to those who were unfamiliar with DoA monitors, those who were familiar with DoA monitors were less likely to report that elderly patients are most in need of DoA monitoring (*p* = 0.025) (Table 2).

In daily clinical practice, a percentage of 72.0% (95%CI 62.6–79.7) of the respondents assessed DoA based on the dosage of anesthetics and vital signs. While only 10.3% (95%CI 5.7–17.7) used a DoA monitor, 11.2% (95%CI 6.4–18.8) used only vital signs, and 6.5% (95%CI 3.1–13.2%) used ETAC. A total of 78.5% (95%CI 69.6–85.3) were satisfied with the DoA achieved in clinical practice and 21.5% (95%CI 14.7–30.4) were dissatisfied. In addition, 90.7% (95%CI 83.5–95.4) agreed that inhaled anesthetics are useful in preventing intraoperative awareness.

Many crucial factors influenced the use of DoA monitors. Inability to bill insurance or high cost (57.0%; 95%CI 47.4–66.1) was the main influencing factor, followed by limited accuracy (30.8%; 95%CI 22.7–40.3), limited sensitivity (29.9%; 95%CI 21.9–39.3), and inability to monitor analgesia (29.9%; 95%CI 21.9–39.3). Furthermore, most of the respondents (85.1%; 95%CI 76.9–91.2) agreed that DoA monitors can prevent awareness, and only 6.5% (95%CI 3.1–13.2) disagreed with this statement. Moreover, 53.3% (95%CI 43.7–62.6) of the respondents considered DoA monitoring more effective than ETAC in preventing awareness, 18.7% (95%CI 12.3–27.3) thought there was no difference, and 8.4% (95%CI 4.4–15.5) considered that DoA monitoring was inferior to ETAC monitoring.

About half of the respondents (50.5%; 95%CI 40.6–60.3) agreed that prolonged low DoA readings (< 40) are associated with adverse outcomes. Additionally, most of the respondents (64.5%; 95%CI 54.6–73.5) agreed to deliver fewer anesthetics with prolonged lower DoA readings (≤ 35). In comparison, 61.7% (95%CI 51.8–70.9) of the respondents agreed to increase anesthetic delivery with prolonged elevated DoA readings (≥ 65). A percentage of 86.0% (95%CI 77.9–91.4) reported that DoA monitoring could reduce the use of anesthetics. A substantial proportion of the respondents (91.6%; 95%CI 84.6–96.1) agreed that DoA monitoring should be mandatory during total intra**v**enous anesthesia (TIVA) with muscle relaxants. In comparison, 85.0% (95%CI 76.9–91.2) of the respondents agreed that DoA monitoring should be mandatory during TIVA without muscle relaxants.

Table 3 shows the current practice of using a DoA monitor and the comparison between those who had a case of intraoperative awareness and those who had not. The most frequently used DoA monitors by our respondents were the BIS monitor (56.1%; 95%CI 46.5–65.3), auditory evoked potential (AEP) (13.1%; 95%CI 7.9–21.0), and patient state index (PSI) (8.4%; 95%CI 4.4–15.5). Compared to those who never had a case of awareness, those who had a case of awareness were more likely to use an AEP monitor (Table 3). Most of the respondents (66.4%; 95%CI 56.8–74.7) believed that the BIS monitor is the most accurate instrument available, followed by the AEP (13.1%; 95%CI 7.9–21.0). Slightly more than half of the respondents (52.3%; 95%CI 42.8–61.7) believed that number, EEG trace, and burst suppression ratio are equally important indicators of the DoA monitors. This did not differ between those who had and had not a case of awareness. In individual practice, DoA monitoring was routinely used by only 6.5% (95%CI 3.1–13.2) of the respondents; it was used in more than one-third of cases by 12.2% (95%CI 7.1–19.9), in less than one-third of cases by 67.3% (95%CI 57.8- 75.6), and never by 14.0% (95%CI 8.6–22.1). This proportion did not differ between those who had and had not a case of awareness (Table 3).

Based on the current DoA monitors used, the respondents believed that the DoA monitoring functions that required further improvement were as follows: accuracy (76.6%; 95%CI 67.6–83.8), suitability for patients of all ages (64.5%; 95%CI 54.9–73.0), all anesthetics (61.0%; 95%CI 47.4–66.1), and analgesia (41.1%; 95%CI 32.-50.8). Almost 40% thought that DoA monitors should be simple and combine EEG and vital sign monitoring. However, less than 20% of the respondents (18.7%; 95%CI 12.3–27.3) believed that advanced DoA monitors should have the strong anti-interference ability and use artificial intelligence (41.1%; 95%CI 32.-50.8). Regarding the application of DoA monitoring and whether DoA monitoring has become as crucial as electrocardiogram (ECG) monitoring in clinical practice, 51.4% (95%CI 41.9–60.8) of the respondents reported a significant difference between the importance of DoA monitoring and that of ECG monitoring.

During 2021, the total number of anesthesia procedures performed under the care of all respondents was 57,833. During 2021, the total number of new instances of accidental awareness during anesthesia that all respondents dealt with was 227 events. Thus, the event rate among our respondents was 0.4% (95%CI 0.34–0.45) which corresponds to 4 intraoperative awareness events for every 1000 procedures. A percentage of 57.5% (95%CI 50.8–64.1) of respondents reported that they faced a case of awareness during surgery. However, less than 20% (17.7%; 95%CI 13–23.3) had exposed to physical or psychological hurt. Based on the characteristics of intraoperative awareness, it was found that more than one-third of respondents (37.6%; 95%CI 31.3- 44.3) stated that awareness events were ascertained only on questioning and only 4.9% (95%CI 2.5–8.5) reported that these events resulted on a formal complaint to hospitals (Table 4).

## Discussion

This survey intended to provide insights into the perception and practice of Jordanian anesthesiologists toward using DoA monitors and estimate the event rate of intraoperative awareness among them. Our results show that although most of the Jordanian anesthesiologists believed that currently, available DoA monitors are effective for DoA monitoring, one-third of them had never used and were unfamiliar with DoA monitors. We found that a higher frequency of using a DoA monitor was associated with a higher education and specialty level, longer experience, and being working for the private sector. In addition, the rate of intraoperative awareness was estimated as four cases for every 1000 anesthesia procedures among anesthesiologists. However, most involved hospitals had no policies to regulate DoA monitor and prevent intraoperative awareness.

Our results are the first to support the need for further investment in DoA monitoring advocacy in developing countries setting where resources are scarce and technology implementation is a barrier to ensuring patient safety practices. The percentage of respondents who routinely used DoA monitors in Jordan (6.5%) was lower than those in China (9.1%), the UK (22.0%), [22] and Australia (53.0%) [23]. One possible explanation is that the availability of DoA monitors is limited in the anesthesia departments in Jordan, particularly in the public hospitals where a higher percentage of anesthesiologists had never used a DoA monitor. This may further be coupled with the limitations in health care insurance policies in Jordan that may not involve DoA monitors as their use is not mandatory by most guidelines and the inability to ensure the cost-effectiveness of DoA monitors [24]. This is evident by our results that the most crucial factor that influenced the use of DoA monitors was the inability to bill or high cost. This can also explain why most Jordanian anesthesiologists assessed DoA using the dosage of anesthetics and vital signs.

Other crucial factors affecting the use of DoA monitors by Jordanian anesthesiologists were limited accuracy, limited sensitivity, and inability to monitor analgesia. These factors were also reported by the Chinese anesthesiologists, (ref) Although most of our respondents (> 80%) found DoA monitors are effective in preventing intraoperative awareness, only one-third of the respondents found DoA monitors accurate or sensitive. In addition, three-fourths of the respondents believed that the accuracy of DoA monitors required further improvement. This may be reflective of the limitations of the available DoA monitors.

Our results indicate that the suitability of DoA monitors for patients of all ages is the second crucial demand reported. More than half of our respondents reported that DoA monitoring was suitable for elderly and adult patients but only 30% reported the suitability of DoA monitors for young children and infants. This finding might be relevant given the limitations of the available DoA monitors when applied to pediatric patients [25]. Several age-related changes factors can influence the readings the DoA monitors in children who frequently have burst-suppressed and discontinuous electroencephalography activity during anesthesia [26]. However, there are DoA monitors that perform well in children, but anesthesiologists should be trained on the specific device applied with its own technology, to make the right interpretation and action.

In addition to the prevention of intraoperative awareness, most respondents believed that the primary purposes of using DoA monitors were to the guide delivery of anesthesia and reduce recovery time. These results are supported by Chinese and Australian studies [19, 20, 23]. Furthermore, many respondents agreed that depth of DoA monitoring should be mandatory during TIVA with and without muscle relaxants. Similar findings were reported in a Chines study [20]. This is in line with the current guidelines that recommend DoA monitoring when using TIVA with neuromuscular blockade whereas it should be considered when TIVA is used alone [19, 20, 22–24, 27–29].

Regarding the usage of DoA monitors, most of our respondents reported that the BIS was the most accurate and frequently used DoA monitor. Similar findings were reported by most anesthesiologists in China, Australia, and Europe [19, 20, 27]. This consistency between countries corresponds with several studies that have documented the superiority of BIS to reduce intraoperative awareness, general anesthetic consumption, and anesthetic recovery times among other types of DoA monitors [28, 29]. However, most anesthesiologists thought that all DoA monitors have limited accuracy and suitability for all anesthetic agents and analgesia. These limitations have been noticeably resolved by using deep learning algorithms that combine EEG signals and multiple vital signs and other exogenous information [30, 31].

The event rate of intraoperative awareness among Jordanian anesthesiologists was relatively high (4/1000 cases) compared to the international context (1–2/1000) [2–4, 19]. The findings of the current study may explain the higher rate of intraoperative awareness events in Jordan. These include the underuse of DoA monitoring, limited knowledge and practices among anesthesiologists, and unavailability of policies aiming to minimize or prevent awareness incidents. However, a more relevant study design is needed to accurately estimate the incidence rate of intraoperative awareness in Jordan.

This study generated several recommendations that should be taken in consideration. These are mainly related to enhancing the anesthesiologist’s practice, knowledge, and attitudes toward intraoperative awareness and DoA monitors. It may be helpful to conduct relevant continuing education and training sessions about the importance and variability of current DoA monitors. We also recommend all hospitals in Jordan adopt new advanced and applicable DoA monitors as routine practice using artificial intelligence. Moreover, healthcare managers and policymakers should focus on managing factors associated with the limited use of DoA monitors and the high rate of intraoperative awareness. All hospitals should consider policies to regulate the use of DoA monitors and prevent intraoperative awareness.

### Limitations

There are several limitations should be considered during the interpretation of our findings. First, the sample size is relatively small and might not be representative. Still, healthcare-related studies usually have such issues. However, our results could be a pilot assessment to investigate a critical healthcare-related practice within a subspecialty. Second, the current study used a convenience sampling method to select participants. A more robust sampling method such as random or cluster sampling might be me necessary to draw a representative sample. Third, this was a self-reported survey and was subjected to reporting bias. Finally, the survey was adapted from studies conducted in developed countries that may not necessarily be applicable to the developing healthcare system in Jordan.

## Conclusion

The estimated event rate of intraoperative awareness in Jordan seems to be high compared with previous international statistics. This is likely due to multiple factors such as the underuse of DoA monitors, inadequate perception and limited knowledge of anesthesiologists, and lack of policies and guidelines concerning the prevention of intraoperative awareness and use of DoA monitors. Large efforts are needed to enhance the perception of anesthesiologists and their practice toward the use of DoA monitors in daily practice. Moreover, healthcare administrators and policymakers should adopt policies and guidelines to prevent intraoperative awareness and regulate the use of DoA monitors. These interventions may eventually reflect positively on patient safety and the quality of healthcare.

## Data Availability

Data will be available upon reasonable request from corresponding author.

## Abbreviations

DoA: Depth of anesthesia
PTSD: Post-traumatic stress disorder
EEG: Electroencephalogram
BIS: Bispectral index
ETAC: End-tidal anesthesia concentration
IRB: Institutional review board
SD: Standard deviation
PSI: Patient state index
AEP: Auditory evoked potential
CSI: Cerebral state index
TIVA: Total intra**v**enous anesthesia
